# Draft genome dataset of *Burkholderia multivorans* sequence type 880, isolated from imported whiteleg shrimp (*Litopenaeus vannamei*) in Norway

**DOI:** 10.1016/j.dib.2026.112807

**Published:** 2026-04-27

**Authors:** Didrik H. Grevskott, Julia E. Storesund, Manish P. Victor, Bjørn Tore Lunestad, Nachiket P. Marathe

**Affiliations:** Department of Contaminants and Biohazards, Institute of Marine Research (IMR), Bergen, Norway

**Keywords:** Norway, Imported seafood, Whiteleg shrimp, *Burkholderia multivorans*, Whole-genome sequencing

## Abstract

Widely distributed in soil and water, *Burkholderia multivorans* is an opportunistic pathogen that most notably affects individuals with cystic fibrosis. The aim of this study was to describe the draft genome sequence of a *B. multivorans* strain that was isolated from a whiteleg shrimp (*Litopenaeus vannamei*) imported to Norway from Vietnam. Genomic DNA was extracted from the isolate, and sequencing was performed, using Illumina MiSeq platform. The isolate belonged to sequence type (ST) 880 and carried multiple beta-lactamases (PEN-A23, AmpC, OXA-1043), as well as several virulence factors, including biofilm formation (*adeG*), toxin production (*hlyB, hlyD*) and secretion systems (*vgrG, tli4, hecB*), suggesting a pathogenic potential. This isolate represents an opportunistic pathogen transported across continents via imported seafood and will serve as an important resource for researchers working on seafood safety.

Specifications Table

**Table:** 

Subject	Biology
Specific subject area	Microbiology and genomics
Type of data	Whole-genome sequencing, Genome Assembly, Annotation.
Data collection	Twenty-seven samples of imported seafood collected at Norwegian border control stations as part of the national surveillance of imported seafood in 2020 and 2021 were examined. One sample of whiteleg shrimp (*L. vannamei*) tested positive for cefotaxime-resistant *B. multivorans*. Genomic DNA was extracted from the isolate, using the DNeasy Blood and Tissue kit (Qiagen, Germany) following the manufacturer’s protocol. Extracted genomic DNA was quantified, using Qubit™ 2.0 Fluorometer with the dsDNA BR (Broad-Range) kit. Sequencing libraries were prepared, using Nextera DNA Flex Library Prep kit (Illumina, USA). Sequencing was performed, using Illumina MiSeq platform (Illumina, USA), with 2 × 300 bp paired-end reads, at the Norwegian Sequencing Centre (Ullevål University Hospital, Oslo, Norway).
Data source location	Latitude: 60.39 °N; Longitude: 5.32 °E; City/Town: Bergen; Country: Norway
Data accessibility	The assembled draft genome sequence of *B. multivorans* 1–1067 has been submitted to DDBJ/ENA/GenBank under the following accession number: JBNCAK000000000. Raw sequence data have been deposited in the Sequence Read Archive under the BioProject accession no PRJNA1250253. Direct URL to SRA data: https://www.ncbi.nlm.nih.gov/sra/?term=SRR37369403
Related research article	None

## 1 Value of the Data

• This dataset provides the first draft genome sequence of a *Burkholderia multivorans* strain, isolated from imported whiteleg shrimp (*Litopenaeus vannamei*) in Norway.
• This strain will serve as an important reference for researchers working on pathogens belonging to the genus *Burkholderia* from seafood.

## 2 Background

*Burkholderia multivorans*, belongs to the *B. cepacia* complex, a group of closely related species recognized for their environmental adaptability and clinical importance [1]. Widely distributed in soil and water, *B. multivorans* is an opportunistic pathogen that most notably affects individuals with cystic fibrosis, where it can colonize the respiratory tract and contribute to chronic lung infection [2]. The organism is characterized by intrinsic resistance to multiple antibiotics, biofilm-forming capacity, and the ability to persist in hostile environments, making infections difficult to eliminate [1]. Although *Burkholderia* spp. are not a common cause of foodborne illness compared to *Vibrio* spp. or *Salmonella* spp. [3], the risk of *Burkholderia* spp. in seafood is generally linked to contamination during processing, handling or water exposure. This study describes the draft genome sequence of a *B. multivorans* sequence type (ST) 880 that was isolated from a whiteleg shrimp (*Litopenaeus vannamei*) imported to Norway from Vietnam.

## 3 Data Description

Briefly, genomes were annotated, using the Prokaryotic Genome Annotation Pipeline (PGAP) v.4.13 at the National Centre for Biotechnology Information (NCBI) [4]. Average Nucleotide Identity values based on BLAST (ANIb) [5] were calculated, using the server JSpeciesWS [6], to confirm the species identity. The assembled genome had a total length of 6,516,535 bp, a G + C content of 67.1% and consisted of 482 contigs (> 500 bp; average coverage, 92 ×), the genome has 6145 coding DNA sequences, 15 rRNAs, and 63 tRNAs. Sequence types (STs) were identified, using the PubMLST database [7], while the presence of antibiotic resistance genes was analyzed, using ResFinder v.4.7.2 [8]. The presence of virulence genes was examined, using the VFanalyzer at the VFDB [9]. The isolate belonged to sequence type (ST) 880 [10] and carried multiple beta-lactamases belonging to class A (PEN-A23), C (AmpC) and D (OXA-1043), explaining the observed resistance to penicillins and cephalosporins. This isolate also carried several virulence factors, including biofilm formation (*adeG*), toxin production (*hlyB, hlyD*) and secretion systems (*vgrG, tli4, hecB*), suggesting a pathogenic potential. This isolate represents an opportunistic pathogen transported across continents via imported seafood and will serve as an important resource for researchers working on seafood safety (Table 1).

**Table 1:** Minimum inhibitory concentrations (MICs) of different antibiotics (mg/l) for *Burkholderia multivorans* isolate 1-1067, isolated from imported whiteleg shrimp (*Litopenaeus vannamei*).

AMP	MERO	CIP	AZI	AMI	GEN	TGC	TAZ	FOT	CHL	COL	NAL	TET	TMP	SMX
> 32	2	0.5	32	< 4	16	< 0.25	4	> 4	< 8	> 16	< 4	< 2	2	16

AMP; Ampicillin, MERO; Meropenem, CIP; Ciprofloxacin, AZI; Azithromycin, AMI; Amikacin, GEN; Gentamicin, TGC; Tigecycline, TAZ; Ceftazidime, FOT; Cefotaxime, CHL; Chloramphenicol, COL; Colistin, NAL; Nalidixic acid, TET; Tetracycline, TMP; Trimethoprim, SMX; Sulfamethoxazole.

## 4 Experimental Design, Materials and Methods

Twenty-seven samples of imported seafood collected at Norwegian border control stations as part of the national surveillance of imported seafood in 2020 and 2021 were examined [11]. Each sample consisted of approximately 750 g of raw, frozen whiteleg shrimp. In order to screen for cefotaxime (CTX)-resistant Enterobacterales, 20 g sample was transferred to 50 ml of MacConkey (MAC) broth (Oxoid, UK) containing 2 mg/l CTX (Sigma-Aldrich, Germany) and incubated overnight at 37 °C, following a modified version of the ISO 6579–1:2017 method (using MAC broth instead of buffered peptone water). After incubation, 10 µl of each enrichment broth were transferred and spread on MAC agar (Oxoid, UK) containing 2 mg/l CTX (Sigma-Aldrich, Germany) and incubated overnight at 37 °C. Pink colonies were identified, using matrix-assisted laser desorption ionization-time of flight mass spectrometry (MALDI-TOF MS) (Bruker Daltonics, Germany) [12]. While targeting CTX-resistant Enterobacterales, one *B. multivorans* isolate from a sample of whiteleg shrimp (*L. vannamei*) was obtained. The resistance profile of this isolate was determined against 15 antibiotics, using a broth microdilution assay with Sensititre® EUVSEC3 plates (Thermo Scientific, USA), following manufacturer’s protocol as previously described [13].

Genomic DNA was extracted from the isolate, using the DNeasy Blood and Tissue kit (Qiagen, Germany) following the manufacturer’s protocol. Extracted genomic DNA was quantified, using Qubit™ 2.0 Fluorometer with the dsDNA BR (Broad-Range) kit. Sequencing libraries were prepared, using Nextera DNA Flex Library Prep kit (Illumina, USA). Sequencing was performed, using Illumina MiSeq platform (Illumina, USA), with 2 × 300 bp paired-end reads, at the Norwegian Sequencing Centre (Ullevål University Hospital, Oslo, Norway).

## Limitations

Although the study is based on a single isolate rather than a longitudinal design, it will serve as an important reference for researchers working on pathogens belonging to the genus *Burkholderia* from seafood. It will allow studying temporal changes within the species with regards to acquired resistance in *Burkholderia* spp. transported across countries via seafood. Depositing this genome sequence into an existing databank would enable its use in core genome analysis, such as single nucleotide polymorphism (SNP)-based approaches, to perform comparative studies of *Burkholderia* spp. strains from different sources and countries.

## Ethics Statement

The authors have read and follow the ethical requirements for publication in *Data in Brief* and confirming that the current work does not involve human subjects, animal experiments, or any data collected from social media platforms.

## Credit Author Statement

**Didrik H. Grevskott**: Methodology, Investigation, Formal analysis, Visualization, Writing – original draft; **Julia E. Storesund**: Resources, Project administration, Funding acquisition, Writing – review & editing; **Manish P. Victor**: Formal analysis, Data curation, Writing – review & editing; **Bjørn Tore Lunestad**: Resources, Funding acquisition, Writing – review & editing; **Nachiket P. Marathe**: Conceptualization, Methodology, Investigation, Validation, Writing – review & editing.

## Data Availability

NCBI SRAAssembled draft genome sequence of B. multivorans 1–1067 (Original data).
